# Comparative Analysis of Scrub Typhus Rapid Kits and Their Relevance in Screening and Diagnosis

**DOI:** 10.7759/cureus.85413

**Published:** 2025-06-05

**Authors:** Viyatprajna Acharya, Shubhransu Patro, Kavita Aggarwal, Rajesh K Dash, Sibabratta Patnaik, Basanti K Pathi

**Affiliations:** 1 Biochemistry, Kalinga Institute of Medical Sciences, Bhubaneswar, IND; 2 General Medicine, Kalinga Institute of Medical Sciences, Bhubaneswar, IND; 3 Microbiology, Kalinga Institute of Medical Sciences, Bhubaneswar, IND; 4 Pediatric Intensive Care Unit, All India Institute of Medical Sciences (AIIMS) Bibinagar, Bibinagar, IND

**Keywords:** cost-effectiveness analysis, endemic region, igm, immunochromatography, odisha, rapid diagnostic test, resource-limited settings, sensitivity, serum igg, specificity

## Abstract

Background: Rapid diagnostic tests (RDTs) are increasingly recognized as practical tools for the early detection of infectious diseases, particularly in resource-limited settings. These tests offer the advantage of portability, ease of use, and quick turnaround times, making them ideal for point-of-care diagnostics. This study evaluates the diagnostic performance of three commercially available RDTs for scrub typhus, using IgM and IgG enzyme-linked immunosorbent assays (ELISAs) as reference standards, to assess their reliability and applicability in clinical and field settings.

Methods: A cross-sectional study was conducted at the Kalinga Institute of Medical Sciences, Eastern India, over one year. Serum samples from 300 clinically suspected scrub typhus cases were tested using three RDTs, with IgM and IgG ELISAs as reference tests. Diagnostic accuracy metrics, such as sensitivity and specificity, were calculated. Statistical analysis compared results across RDTs.

Results: Of the 300 samples, 125 (41.67%) tested positive for scrub typhus by ELISA. For IgM detection, sensitivities ranged from 89.60% to 96.80% and specificities from 84.00% to 98.29%. For IgG, sensitivities ranged from 51.06% to 74.47% and specificities from 82.21% to 94.47%. The differences in diagnostic performance among RDTs were statistically significant (P < 0.05). Clinical parameters indicated significant differences in direct bilirubin, total protein, albumin-to-globulin (A:G) ratio, and potassium levels between the scrub typhus-positive and negative groups.

Conclusions: This research is important and applicable, considering the widespread occurrence of scrub typhus in India and the demand for easy-to-use diagnostic tools in areas with limited resources. It confirms that RDTs are a practical, IgM-based tool for early scrub typhus detection owing to their ease of use and portability, suiting point-of-care needs, but quality control and validation are crucial. Positive correlation with inflammatory markers further underscores its utility as a diagnostic tool. Further multicenter, longitudinal, and cost-effectiveness studies are needed to support their wider adoption in national programs.

## Introduction

The accurate and timely diagnosis of infectious diseases is crucial for effective disease management and public health interventions. Various laboratory methods, including culture-based method, polymerase chain reaction (PCR), immunofluorescence assay (IFA), enzyme-linked immunosorbent assay (ELISA), and electrochemiluminescence immunoassay (ECLIA), are employed for diagnosis, each with distinct principles, advantages, and limitations [1-4]. Culture-based method is highly specific but impractical due to its time-consuming nature and the need for specialized laboratory infrastructure. PCR offers high sensitivity and specificity but requires advanced equipment and skilled personnel [5], while IFA, which detects antibodies using fluorescent-labelled antigens, is subject to observer variability. ELISA and ECLIA are widely used serological methods for detecting IgM or IgG antibodies. Although ELISA provides high sensitivity and specificity, it requires a well-equipped laboratory, skilled personnel, and a longer turnaround time, making it less feasible in resource-limited settings [5]. ECLIA provides enhanced sensitivity and automation; however, its high cost limits accessibility in many endemic regions. In contrast to all the above-described diagnostic methods, immunochromatographic (IC) rapid diagnostic tests (RDTs) provide a practical solution for rapid and on-site diagnosis. These tests operate on the principle of antigen-antibody interactions, in which target antibodies present in a patient’s sample bind to immobilized antigens on a nitrocellulose strip, producing a visually interpretable result within 15-20 minutes. RDTs are simple to use, require minimal training, and can be performed without sophisticated laboratory infrastructure.

Given the importance of accurate and accessible diagnostic methods, it is essential to evaluate their application in the context of specific infectious diseases. One such disease is scrub typhus, a vector-borne zoonotic infection transmitted to humans through the bite of infected chigger mites (*Leptotrombidium *spp.). It is endemic to the Asia-Pacific region, commonly referred to as the ‘tsutsugamushi triangle’ [4]. However, reports of cases in Dubai [6], Chile [7], and Kenya [8], as well as its detection in rodent populations in West Africa and Europe, suggest that scrub typhus is no longer confined to the ‘tsutsugamushi triangle’. Recognized by the World Health Organization (WHO) as a neglected disease, scrub typhus remains a significant public health challenge, particularly in the Asia-Pacific region [9]. The causative agent, *Orientia tsutsugamushi*, is an intracellular bacterium that evades host immune responses, making early and accurate diagnosis essential for effective treatment. In India, scrub typhus has emerged as a major public health issue, significantly contributing to acute undifferentiated febrile illnesses (AUFI) with high rates of morbidity and mortality [2,10]. The number of reported outbreaks has increased dramatically, with cases documented across multiple states, including Andhra Pradesh, Tamil Nadu, Karnataka, Kerala, Assam, West Bengal, Bihar, Himachal Pradesh, and Odisha, among others [11]. Last year, Odisha reported a surge in scrub typhus cases across five districts: Kalahandi, Bargarh, Sundargarh, Keonjhar, and Sambalpur, with eight confirmed deaths in two districts. Sundargarh had over 200 cases, while Keonjhar recorded 630 [12]. Hospitalization due to scrub typhus in AUFI cases accounts for up to 35-50% [10,13,14], and without proper treatment, the case fatality rate (CFR) can rise to 30-70% [11]. A recent meta-analysis revealed regional differences in scrub typhus prevalence across India. The highest rates were observed in the southern states (30.23%), followed by the north-eastern region (20.62%). Although the western part of the country reported a lower prevalence, it showed a significantly high case fatality rate of 33% [15]. A population-based cohort study conducted in rural villages of Tamil Nadu further emphasized the public health significance of the disease, estimating an incidence of 6.0 clinical cases per 1,000 person-years [16]. Sondhiya et al. suggested that the increasing burden may be partly due to the overuse of broad-spectrum antibiotics for treating other febrile illnesses, potentially contributing to both increased prevalence and disease severity. A major limitation in estimating the true burden is the underreporting of febrile illnesses [15]. Nonetheless, these findings confirm that *O. tsutsugamushi* infection is widely endemic in India, underscoring the urgent need for robust surveillance and early, sensitive diagnostic tools to control its spread effectively.

The presence of an eschar is a highly specific diagnostic marker, reportedly present in up to 98.9% of cases. However, eschar presentation varies widely among patients (7-97%) and is rarely observed in Indian cases, necessitating laboratory confirmation [1,5,11]. Given the constraints of traditional methods, including the Weil-Felix test, which relies on cross-reactivity between *O. tsutsugamushi *and *Proteus *antigens but has poor specificity [1,5,17], RDTs represent a promising alternative for rapid and accessible diagnosis. However, differences in diagnostic accuracy among various RDT brands necessitate a thorough comparative assessment to establish their reliability in field conditions.

This study evaluates the diagnostic performance of three commercially available RDT brands widely used in hospitals and health centers across India, in comparison with IgM and IgG ELISAs. The results highlight their potential for large-scale screening programs and aim to establish RDTs as a viable diagnostic tool for endemic areas, supporting their integration into routine disease surveillance and control programs. 

## Materials and methods

Study type and location

**Figure 1:**
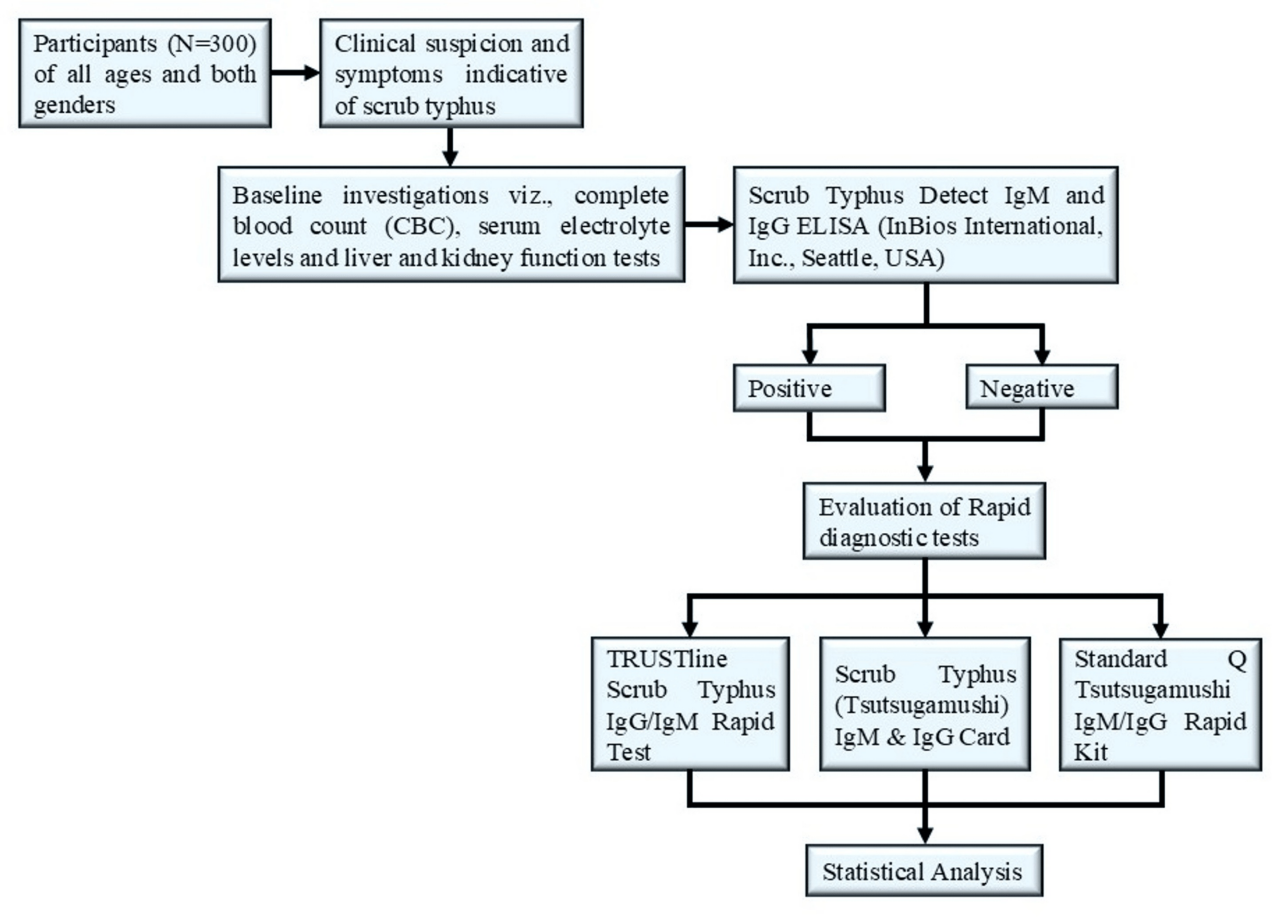
Overview of the study design workflow ELISA: Enzyme-linked immunosorbent assay; RDT: Rapid diagnostic test

This cross-sectional study was carried out at the Kalinga Institute of Medical Sciences, a tertiary care hospital located in Eastern India, in collaboration with the Departments of Biochemistry, Microbiology, Internal Medicine, and Pediatrics. The study was conducted over a period of one year, from December 2022 to December 2023. An overview of the study design workflow is depicted in Figure 1.

Study participants

A total of 300 patients, of all ages and both genders, presenting with clinical suspicion and symptoms indicative of scrub typhus, such as persistent fever, chills, headaches, body aches, rashes on the trunk and extremities, enlarged lymph nodes, and mental confusion, were included in the study.

Inclusion Criteria

Patients presenting with acute febrile illness of ≥5 days duration and at least one additional symptom suggestive of scrub typhus, as listed above, and those willing to provide informed consent were included.

Exclusion Criteria

Patients were excluded if they had a confirmed alternative diagnosis explaining the febrile illness, such as malaria, dengue, leptospirosis, or enteric fever. Additionally, individuals who did not wish to give informed consent were not enrolled.

Laboratory investigations and ELISA testing 

After obtaining informed consent, sociodemographic details were documented, and blood samples were collected aseptically and labelled with anonymized codes. The routine baseline investigations included analyzing the complete blood count (CBC), assessing serum electrolyte levels, and conducting liver and kidney function tests. Scrub typhus diagnosis was confirmed using Scrub Typhus Detect IgM ELISA (InBios International Inc., Seattle, USA) and Scrub Typhus Detect IgG ELISA (InBios International Inc., Seattle, USA). Cut-off values were determined according to the guidelines provided by the kit manufacturers using the formula:

Cut-off value = Average of the normal human sera (NHS) + Three times the standard deviation (SD) of NHS

Samples with OD values exceeding the cut-off were classified as positive, while those with OD values below the cut-off were classified as negative.

Evaluation of RDTs

Three commercially available RDTs for detecting scrub typhus infection were evaluated: TRUSTline Scrub Typhus IgG/IgM Rapid Test (Athenese Dx Pvt. Ltd., Chennai, India), Scrub Typhus (Tsutsugamushi) IgM & IgG Card (J. Mitra and Co. Pvt. Ltd., New Delhi, India), and Standard Q Tsutsugamushi IgM/IgG Rapid Kit (SD Biosensor Healthcare Pvt. Ltd., Gurugram, India). Henceforth, these RDTs will be referred to as Athenese Dx-RDT: TRUSTline Scrub Typhus IgG/IgM Rapid Test; J. Mitra-RDT: Scrub Typhus (Tsutsugamushi) IgM & IgG Card; and SD Biosensor-RDT: Standard Q Tsutsugamushi IgM/IgG Rapid Kit.

These RDTs utilize immunochromatography, where the sample moves across a nitrocellulose membrane through capillary action. If IgG or IgM antibodies are present, they bind to antigen-colloidal gold conjugates in the conjugate pad, forming immunocomplexes. These complexes are then captured by anti-human IgG or IgM antibodies on the test lines, producing burgundy-colored bands. A control line ensures the test's validity by consistently displaying a burgundy band, regardless of the test results. These rapid tests are user-friendly, deliver quick results, and are highly portable, making them ideal for field applications. However, their performance can be influenced by various factors, and inadequate training may increase the risk of false negatives. For instance, insufficient sample volume may prevent the sample from reaching the reaction area, while highly viscous or particulate-laden samples can disrupt membrane flow. Additionally, air bubbles, uneven sample application, or physical obstructions may interfere with the test's accuracy. Environmental conditions, such as extreme temperatures or humidity, can also compromise results. Misreading faint test lines or overlooking the control line may lead to incorrect interpretations. Though these tests require minimal technical expertise, their accuracy and reliability depend on proper training, strict adherence to protocols, and careful execution and interpretation to minimize errors [18].

The RDTs were evaluated using both confirmed positive serum samples (via ELISA) and negative controls. Each sample was tested concurrently with all three RDTs following the respective manufacturers' instructions at room temperature. Personnel performing the RDTs were blinded to the ELISA results to minimize observer bias. Results were read visually within the designated timeframe and recorded as qualitative outcomes, either 'positive' or 'negative'. To minimize subjectivity and inter-observer variability, each test result was independently evaluated by two trained researchers. If there was any disagreement between the initial readers regarding the presence or intensity of the test lines, a third senior researcher was brought in to make the final decision. This researcher was not informed of the earlier interpretations or the clinical details of the patient, ensuring an unbiased assessment. The final reading was made within the timeframe recommended by the manufacturers to maintain accuracy, and this reading was considered definitive for the analysis.

Statistical analysis

The recorded demographic and clinical data of the patients were analyzed using Microsoft Excel (Microsoft Corp., Redmond, USA). To determine whether the data followed a normal distribution, the Shapiro-Wilk test was performed in R version 4.4.2 (R Foundation for Statistical Computing, Vienna, Austria). Descriptive variables were summarized as percentages, mean ± SD, and median with interquartile ranges. Mann-Whitney U test, a non-parametric test, was chosen in R version 4.4.2 to compare the median values between the negative and positive groups [19].

Diagnostic accuracy encompassing sensitivity, specificity, positive predictive value (PPV), negative predictive value (NPV), and agreement between individual RDTs and the reference test was evaluated using MedCalc's Diagnostic Test Evaluation Calculator version 23.0.9 (MedCalc Software Ltd., Ostend, Belgium) [20]. Uncertainty was expressed as 95% confidence intervals (CI). McNemar’s chi-square test was used to compare the paired proportions of positive and negative results between each RDT based on the ELISA results. This test is particularly appropriate for analyzing paired nominal data, where the same samples are assessed using two different diagnostic methods. It helps determine whether there is a statistically significant difference in the number of discordant pairs. By accounting for the matched nature of the data, the test reduces the risk of drawing misleading conclusions that could arise from treating paired results as independent. The analysis was performed using MedCalc's McNemar Test on Paired Proportions version 23.0.9 (MedCalc Software Ltd., Ostend, Belgium) [21].

Ethical considerations

The research protocol for the use of patients' sera in this comparative evaluation study was reviewed and approved by the Institutional Ethics Committee of Kalinga Institute of Medical Sciences (approval number KIIT/KIMS/IEC/911/2022).

## Results

A total of 300 serum samples (102 female, 198 male) were evaluated in this study. The cut-off values for the reference InBios Scrub Typhus Detect IgG and IgM ELISAs were calculated as 0.298 and 0.5, respectively. Of the 300 samples, 125 (41.67%) tested positive for scrub typhus, including 78 (62.40%) IgM-positive and 47 (37.60%) positive for both IgM and IgG, while 175 samples (58.33%) were classified as negative. Clinical parameters were compared between the ELISA-positive and ELISA-negative groups (Table 1), and statistically significant differences (P < 0.05) were observed for direct bilirubin, total protein, albumin-to-globulin (A:G) ratio, and potassium levels.

**Table 1:** Baseline demographic and clinical parameters of ELISA (InBios - Scrub Typhus Detect) characterized patient samples ^a^: Values are expressed as mean ± SD; ^b^: Value is not expressed as mean ± SD; ^c^: Values are expressed as median with interquartile range ELISA: Enzyme-linked immunosorbent assay; TLC: Total leukocyte count; Hb: Hemoglobin; PCV: Packed cell volume; MCV: Mean corpuscular volume; MCH: Mean corpuscular hemoglobin; MCHC: Mean corpuscular hemoglobin concentration; ALP: Alkaline phosphatase; A:G: Albumin-to-globulin; SD: Standard deviation; F: Female; M: Male

Patient Characteristics^c^	Negative (n=175)	Positive (n=125)	P Value
Age^a^	38.91 ± 23.75	32.12 ± 22.00	-
Sex (F/M)^b^	59/116	43/82	-
TLC	8.60 (5.90 - 12.30)	7.61 (5.75 - 10.80)	0.273
Hb, %	10.90 (9.65 - 12.40)	11.20 (9.80 - 13.10)	0.185
PCV, %	34.40 (30.10 - 39.20)	35.30 (32.70 - 40.70)	0.096
MCV, fl	81.40 (75.35 - 87.65)	82.20 (73.30 - 86.30)	0.539
Hematocrit, %	44.2 (38.8 - 48.9)	44.2 (38.8 - 49.5)	0.279
MCH, pg	25.70 (23.60 - 27.95)	25.50 (23.10 - 27.60)	0.537
MCHC, g/dl	31.60 (30.60 - 32.30)	31.60 (30.60 - 32.30)	0.991
Platelet count	197.00 (150.00 - 278.00)	176.00 (150.00 - 224.00)	0.184
Total bilirubin, mg/dl	0.73 (0.47 - 1.15)	0.78 (0.48 - 1.20)	0.521
Direct Bilirubin, mg/dl	0.25 (0.14 - 0.43)	0.20 (0.05 - 0.40)	0.04
ALP, IU/l	102.00 (75.50 - 139.00)	108.00 (79.00 - 147.00)	0.224
Total protein, mg/dl	6.60 (5.90 - 7.30)	7.10 (6.10 - 7.60)	0.012
Albumin, g/dl	3.50 (3.01 - 3.90)	3.40 (2.93 - 4.00)	0.445
A:G ratio	1.10 (0.91 - 1.30)	1.00 (0.78 - 1.21)	0.011
Urea, mg/dl	25.00 (17.00 - 43.00)	29.00 (19.00 - 45.00)	0.119
Creatinine, mg/dl	0.84 (0.55 - 1.19)	0.80 (0.53 - 1.04)	0.466
Procalcitonin, ng/ml	0.67 (0.33 - 1.60)	1.01 (0.30 - 4.02)	0.175
Sodium, mmol/l	134.00 (131.00 - 137.50)	135.00 (131.00 - 139.00)	0.139
Potassium, mmol/l	3.92 (3.50 - 4.40)	4.10 (3.73 - 4.50)	0.047
Chloride, mmol/l	81.0 (76.0 - 87.5)	78.0 (74.0 - 88.0)	0.107

The ELISA reference test identified 47 (15.67%) samples as IgG-positive and 125 (41.67%) as IgM-positive, while 253 (84.33%) samples were IgG-negative and 175 (58.33%) were IgM-negative. These samples were tested using three different RDTs, and the results for IgG and IgM detection, compared to the reference test, are summarized in Tables 2-3. Among the three RDTs, Athenese Dx-RDT detected the highest number of positive samples, identifying 35 (74.47%) IgG-positive samples and 121 (96.80%) IgM-positive samples. The percent agreement with the reference test for IgG detection was 87.33% for Athenese Dx-RDT, 78.67% for J. Mitra-RDT, and 87.67% for SD Biosensor-RDT (Table 2).

**Table 2:** Comparison of the three IgG rapid tests evaluated with the reference test (InBios Scrub Typhus Detect IgG ELISA) for diagnosis of scrub typhus infection Athenese Dx-RDT: TRUSTline Scrub Typhus IgG/IgM Rapid Test; J. Mitra-RDT: Scrub Typhus (Tsutsugamushi) IgM & IgG Card; SD Biosensor-RDT: Standard Q Tsutsugamushi IgM/IgG Rapid Kit ELISA: Enzyme-linked immunosorbent assay; RDT: Rapid diagnostic test

Reference Test (InBios ELISA)	IgG					
	Athenese Dx RDT		J. Mitra RDT		SD Biosensor RDT	
	Positive, n (%)	Negative, n (%)	Positive, n (%)	Negative, n (%)	Positive, n (%)	Negative, n (%)
Positive (n=47)	35 (74.47%	12 (25.53%)	28 (59.57%)	19 (40.43%)	24 (51.06%)	23 (48.94%)
Negative (n=253)	26 (10.28%)	227 (89.72%)	45 (17.79%)	208 (82.21%)	14 (5.53%)	239 (94.47%)
Percent agreement with reference	87.33%		78.67%		87.67%	

**Table 3:** Comparison of the three IgM rapid tests evaluated with the reference test (InBios Scrub Typhus Detect IgM ELISA) for diagnosis of scrub typhus infection Athenese Dx-RDT: TRUSTline Scrub Typhus IgG/IgM Rapid Test; J. Mitra-RDT: Scrub Typhus (Tsutsugamushi) IgM & IgG Card; SD Biosensor-RDT: Standard Q Tsutsugamushi IgM/IgG Rapid Kit ELISA: Enzyme-linked immunosorbent assay; RDT: Rapid diagnostic test

Reference Test (InBios ELISA)	IgM					
	Athenese Dx RDT		J. Mitra RDT		SD Biosensor RDT	
	Positive, n (%)	Negative, n (%)	Positive, n (%)	Negative, n (%)	Positive, n (%)	Negative, n (%)
Positive (n=125)	121 (96.80%)	4 (3.20%)	112 (89.60%)	13 (10.40%)	118 (94.40%)	7 (5.60%)
Negative (n=175)	3 (1.71%)	172 (98.29%)	28 (16.00%)	147 (84.00%)	17 (9.71%)	158 (90.29%)
Percent agreement with reference	97.67%		91.88%		95.76%	

For IgM detection, the percent agreement was observed as 97.67% for Athenese Dx-RDT, 91.88% for J. Mitra-RDT, and 95.76% for SD Biosensor-RDT (Table 3).

For IgG detection, Athenese Dx-RDT demonstrated significantly higher sensitivity (74.47%) and NPV (94.98%) compared to the other RDTs (P < 0.05) (Table 4). However, its specificity (89.72%) and PPV (57.38%) were lower than those of SD Biosensor-RDT (specificity: 94.47%, PPV: 63.16%) but slightly higher than those of J. Mitra-RDT (specificity: 82.21%, PPV: 38.36%). For IgM detection, Athenese Dx-RDT outperformed the other RDTs, demonstrating significantly higher sensitivity (96.80%), specificity (98.29%), PPV (97.58%), and NPV (97.73%) (P < 0.05) (Table 4). It was followed by SD Biosensor-RDT (sensitivity: 94.40%; specificity: 90.29%; PPV: 87.41%; NPV: 95.76%) and J. Mitra-RDT (sensitivity: 89.60%; specificity: 84.00%; PPV: 80.00%; NPV: 91.88%). A significant difference (P < 0.05) was observed between SD Biosensor-RDT and J. Mitra-RDT for IgG detection, while the difference for IgM detection between these two RDTs was not significant (Table 4).

**Table 4:** Performance characteristics of the three diagnostic kits that screen for scrub typhus infection ^a, b, c^: Tests sharing common superscript differ significantly (P < 0.05); *: Tests sharing common superscript differ insignificantly (P > 0.05) Number of samples tested = 300 (IgG Positive = 47, IgM Positive = 125, IgG Negative = 253, and IgM Negative = 175) Athenese Dx-RDT: TRUSTline Scrub Typhus IgG/IgM Rapid Test; J. Mitra-RDT: Scrub Typhus (Tsutsugamushi) IgM & IgG Card; SD Biosensor-RDT: Standard Q Tsutsugamushi IgM/IgG Rapid Kit PPV: Positive predictive value; NPV: Negative predictive value; CI: Confidence interval; ELISA: Enzyme-linked immunosorbent assay; RDT: Rapid diagnostic test

Test	Marker	InBios Scrub Typhus Detect IgG and IgM ELISAs as reference tests, % (95% CI)			
		Sensitivity	Specificity	PPV	NPV
Reference Test (InBios ELISA)	IgG	100	100	100	100
Reference Test (InBios ELISA)	IgM	100	100	100	100
Athenese Dx RDT^a^	IgG	74.47 (59.65 - 86.06)	89.72 (85.31 - 93.18)	57.38 (47.42 - 66.77)	94.98 (92.06 - 96.86)
Athenese Dx RDT^bc^	IgM	96.80 (92.01 - 99.12)	98.29 (95.07 - 99.65)	97.58 (92.92 - 99.20)	97.73 (94.25 - 99.12)
J. Mitra RDT^a^	IgG	59.57 (44.27 - 73.63)	82.21 (76.93 - 86.72)	38.36 (30.39 - 47.00)	91.63 (88.51 - 93.96)
J. Mitra RDT^b^*	IgM	89.60 (82.87 - 94.35)	84.00 (77.71 - 89.10)	80.00 (73.92 - 84.95)	91.88 (87.07 - 95.00)
SD Biosensor RDT^a^	IgG	51.06 (36.06 - 65.92)	94.47 (90.89 - 96.94)	63.16 (48.95 - 75.40)	91.22 (88.57 - 93.31)
SD Biosensor RDT^c^*	IgM	94.40 (88.80 - 97.72)	90.29 (84.90 - 94.24)	87.41 (81.51 - 91.62)	95.76 (91.65 - 97.89)

## Discussion

Scrub typhus remains a significantly neglected tropical disease despite its growing prevalence across many parts of Asia, including India [15,16]. It contributes to substantial morbidity and mortality, particularly in rural and resource-limited settings where access to timely diagnosis and treatment is often inadequate. With climate change, urban expansion, and increased human contact with mite-infested areas, the risk of scrub typhus is rising [22]. In this context, timely and accurate diagnosis is critical, as delays in treatment can lead to serious complications.

Conventional laboratory-based techniques, including PCR, ELISA, and IFA, offer high sensitivity and specificity but are often limited by their reliance on advanced equipment, skilled personnel, and longer processing times. In contrast, RDTs offer a faster and more practical alternative, enabling early diagnosis with minimal infrastructure requirements. However, the diagnostic performance of RDTs can vary significantly between brands, necessitating thorough evaluation to ensure their reliability.

This study assessed the diagnostic performance of three commercially available RDTs by comparing their results with those of the InBios Scrub Typhus Detect IgM and IgG ELISAs, which served as reference standards. In addition, clinical parameters were analyzed between ELISA-positive and ELISA-negative groups. Notably, direct bilirubin levels and A:G ratio were significantly lower in the ELISA-positive group, while total protein and potassium levels were significantly higher compared to the ELISA-negative group. The elevated total protein levels may indicate an acute-phase response or increased immunoglobulin production associated with infection.

Although levels of total bilirubin, alkaline phosphatase (ALP), urea, procalcitonin, sodium, and potassium were also higher in the ELISA-positive group, these differences did not reach statistical significance. Nonetheless, this trend aligns with findings from other studies conducted in India [23-26]. For instance, Jamwal et al. reported elevated procalcitonin levels in most scrub typhus patients [26], a pattern consistent with our observation of higher procalcitonin concentrations in the ELISA-positive group compared to the ELISA-negative group.

Among the three RDTs evaluated, Athenese Dx-RDT demonstrated the highest performance for IgM detection (P < 0.05), with a sensitivity of 96.80% and a specificity of 98.29%. J. Mitra-RDT showed good sensitivity (89.60%) but a lower specificity (84.00%), raising concerns about false positives in clinical use. SD Biosensor-RDT had balanced sensitivity (94.40%) and specificity (90.29%) but did not outperform Athenese Dx-RDT on either metric. For IgG detection, Athenese Dx-RDT identified the highest number of true positives (74.47%), suggesting superior sensitivity; however, it also exhibited a moderate number of false positives, resulting in slightly lower specificity (89.72%) compared to SD Biosensor-RDT (94.47%). Interestingly, SD Biosensor-RDT, despite demonstrating the highest specificity, had the lowest sensitivity (51.06%), indicating that it may miss a substantial proportion of true IgG-positive cases. J. Mitra-RDT fell between the two in terms of sensitivity (59.57%) but had the lowest specificity (82.21%), potentially leading to more false-positive results. Due to limited data availability, a direct comparison of Athenese Dx-RDT and J. Mitra-RDT with published results was not possible. However, the observed sensitivity and specificity of SD Biosensor RDT for both IgG and IgM closely align with those reported in a previous validation study of the same kit [27]. These trends indicate that while Athenese Dx-RDT offers strong overall performance for both IgM and IgG, trade-offs exist between sensitivity and specificity across the RDTs, which should be carefully considered based on clinical and epidemiological priorities.

The differences in diagnostic performance observed among the RDTs may, in part, be explained by variations in their manufacturing processes, particularly in antigen selection, antibody quality, and overall test design. Kits that incorporate regionally relevant antigens or high-affinity monoclonal antibodies targeting circulating *O. tsutsugamushi* strains are likely to demonstrate improved sensitivity. In contrast, reduced sensitivity may result from the use of antigens with limited cross-strain recognition. However, a detailed comparison remains challenging due to the lack of publicly available information on the specific manufacturing and validation protocols employed by each kit.

The differences in the diagnostic utility of IgM and IgG further highlight the complexity of scrub typhus diagnosis. IgM is a more reliable indicator of recent infection, while IgG, which can remain elevated for months or years, may signal past exposure. In fact, studies in India have shown that IgM can stay above diagnostic levels for up to a year and IgG for more than 36 months [28], much longer than what was observed in similar studies from South Korea [29]. Varghese et al. suggested that these variations may be influenced by genetic and immunological differences among human populations or bacterial strain variations across endemic regions [28]. Therefore, relying solely on either IgM or IgG may lead to misinterpretation of infection status, emphasizing the need for a more comprehensive diagnostic approach.

Given these challenges, combining IgM and IgG testing with clinical evaluation enhances diagnostic accuracy. This integrated approach helps distinguish between recent and past infections, minimizes false positives or negatives from individual antibody tests, and reduces reliance on potentially unreliable patient history. By providing a clearer picture of infection status, it ensures a more precise diagnosis and facilitates the prompt initiation of appropriate antibiotic therapy, which is essential for reducing complications in scrub typhus. This improved clinical decision-making also supports more effective public health responses in endemic regions.

However, the variability in RDT performance remains a concern. As highlighted in a meta-analysis by Saraswati et al., the same RDT brand can yield different results depending on the settings [5], underscoring the importance of consistent quality control and standardized testing protocols. Stephen et al. also emphasized that regular quality checks, including daily testing with both negative controls and positive controls at low and moderate analyte levels, are crucial for ensuring reliable results [30].

The sensitivity and specificity of RDTs can be improved through various strategies. These include using advanced detection labels such as quantum dots and up-conversion fluorescent nanoparticles, incorporating highly specific recognition elements like specific monoclonal antibodies or aptamers to reduce cross-reactivity, and developing more sophisticated readers. For instance, the use of surface-enhanced Raman spectroscopy (SERS) has shown promise in significantly boosting the sensitivity of RDTs [31].

Considering their suitability for mass screening, RDTs have strong potential as frontline diagnostic tools in endemic regions. However, their broader implementation must be supported by stringent quality assurance protocols and ongoing validation. Multicenter studies are essential to assess consistency across diverse epidemiological settings, and longitudinal data would help evaluate test performance over the course of illness and recovery. Additionally, cost-effectiveness studies are vital for determining the economic feasibility of widespread RDT deployment, especially in resource-constrained environments.

While informative, this study has certain limitations. It was conducted at a single tertiary care center, which may not fully represent the diagnostic performance of RDTs across other endemic regions with varying epidemiological patterns, healthcare infrastructures, or circulating *O. tsutsugamushi *strains. Such geographical and strain-based variability could significantly influence test sensitivity and specificity. Furthermore, the absence of longitudinal data precluded the evaluation of antibody kinetics and test performance over the course of illness and recovery. This study also did not explore how test results might correlate with disease severity or evaluate the potential for cross-reactivity with other febrile illnesses.

## Conclusions

This study confirms that RDTs are a practical alternative to conventional tests for scrub typhus, particularly for early IgM-based detection. Their simplicity and portability make them ideal for point-of-care use in low-resource settings. Integrating RDTs into national diagnostic and surveillance programs could enhance early case identification, enable timely treatment, and alleviate the burden on tertiary healthcare systems. However, to support broader implementation, rigorous quality control measures and standardized interpretive guidelines must accompany their deployment.

Future research should prioritize multicenter validation studies to assess the consistency of RDT performance across diverse geographic and epidemiological settings. Longitudinal studies are essential to elucidate the kinetics of IgM and IgG antibodies and to evaluate diagnostic performance throughout the course of illness and recovery. Additionally, cost-effectiveness analyses would provide critical insights to inform policy decisions regarding large-scale RDT implementation. Collectively, these efforts can strengthen the diagnostic landscape for scrub typhus and contribute to more effective surveillance and disease control strategies.
